# The role of hermaphrodites in the experimental evolution of increased outcrossing rates in *Caenorhabditis elegans*

**DOI:** 10.1186/1471-2148-14-116

**Published:** 2014-06-02

**Authors:** Sara Carvalho, Ivo M Chelo, Christine Goy, Henrique Teotónio

**Affiliations:** 1 Instituto Gulbenkian de Ciência, P-2781-901 Oeiras, Portugal; 2 École Normale Supérieure, Institut de Biologie de l’ENS (IBENS), and Inserm U1024, and CNRS UMR 8197, F-75005 Paris, France

## Abstract

**Background:**

Why most organisms reproduce via outcrossing rather than selfing is a central question in evolutionary biology. It has long ago been suggested that outcrossing is favoured when it facilitates adaptation to novel environments. We have previously shown that the experimental evolution of increased outcrossing rates in populations of the male-hermaphrodite nematode *Caenorhabditis elegans* were correlated with the experimental evolution of increased male fitness. However, it is unknown whether outcrossing led to adaptation, and if so, which fitness components can explain the observed increase in outcrossing rates.

**Results:**

Using experimental evolution in six populations with initially low standing levels of genetic diversity, we show with head-to-head competition assays that population-wide fitness improved during 100 generations. Since outcrossing rates increased during the same period, this result demonstrates that outcrossing is adaptive. We also show that there was little evolution of hermaphrodite fitness under conditions of selfing or under conditions of outcrossing with unrelated tester males. We nonetheless find a positive genetic correlation between hermaphrodite self-fitness and population-wide fitness, and a negative genetic correlation between hermaphrodite mating success and population-wide fitness. These results suggest that the several hermaphrodite traits measured are fitness components. Tradeoffs expressed in hermaphrodites, particularly noticed between self-fitness and mating success, may in turn explain their lack of change during experimental evolution.

**Conclusions:**

Our findings indicate that outcrossing facilitates adaptation to novel environments. They further indicate that the experimental evolution of increased outcrossing rates depended little on hermaphrodites because of fitness tradeoffs between selfing and outcrossing. Instead, the evolution of increased outcrossing rates appears to have resulted from unhindered selection on males.

## Background

A central problem in evolutionary biology is to understand why the majority of organisms outcross when reproduction by selfing is in principle more advantageous. Selfers do not suffer the cost of having male offspring that cannot have offspring on their own [1-3], and selfing lineages always segregate more homozygous progeny, which in large populations allows selection to prevent the build-up of the genetic loads responsible for inbreeding depression [4-6]. Also, selfers might not suffer from the physiological problems associated with mating with another individual, such as those involved in copulation or in mate searching [7,8], or from demographic costs when colonizing new habitats on their own [9,10].

August Weissman suggested that increased fitness variance in sexual lineages would favour their maintenance over asexual lineages [11]. Analogously, maintenance of outcrossing versus selfing may be due to increased fitness variance in populations with higher outcrossing rates [12,13]. For example, it has been suggested that in small populations, higher inbreeding depression due to deleterious recessive alleles can be endured with outcrossing than with selfing [4,5]. In larger sized populations, sexual selection may be involved in removing deleterious recessive alleles [14,15]. Outcrossing may also increase effective recombination rates and allow the creation of genotypes that could be crucial for adaptation to novel environments or to resist rapidly adapting pathogens [16,17].

It has been difficult to demonstrate how outcrossing rates evolve as a function of the fitness components expressed under selfing and under outcrossing [18-21]. One of the problems is to determine the sign and degree of genetic correlations among fitness components because they depend on population history and environmental context. In this regard, experimental evolution has long been a favourite approach to determine the nature of genetic correlations among fitness components, for example by measuring correlated trait changes to different selection regimes; e.g., [22,23]. However, when populations with standing genetic diversity face novel environments positive genetic correlations among fitness components are expected. This is because genotype-by-environment interactions initially lead to the fixation/removal of alleles affecting all fitness components, before antagonistic pleiotropic alleles generate variation in fitness [24,25]. In contrast, experiments with populations without initial standing genetic diversity give an unbiased picture of the genetics of fitness components, despite history and environmental context, as they depend on mutational input for evolution [26,27].

Here, we characterize the experimental evolution of fitness in *Caenorhabditis elegans* populations and test how selfing and outcrossing fitness components determine adaptation. For this, we use *C. elegans* populations with little initial standing genetic diversity and take advantage of their natural mixed (androdioecious) reproduction system, where hermaphrodites self and only outcross when mated by males [28]. We have previously shown that in the experimental populations used here initially rare males reached a frequency of 15% after 100 generations of evolution, concurrently with a 100% increased male fitness during the same period [29].

Using several head-to-head competition assays, we find that population-wide fitness increased during 100 generations of experimental evolution, thus indicating that outcrossing is adaptive in our laboratory environment. We further show that hermaphrodites may have improved their fitness when selfing but not when outcrossing, suggesting that increased outcrossing rates are not invariably adaptive. Analysis of genetic correlations among fitness components suggests that hermaphrodite self-fitness and hermaphrodite mating success determined adaptation together with male fitness. However, and because hermaphrodites cannot maximize their fitness under both selfing and outcrossing, we conclude that males drove increased outcrossing rates during experimental evolution.

## Results

### Adaptation: evolution of population-wide fitness

To determine fitness changes during experimental evolution, we contemporaneously measured the competitive performance of the six replicate populations against a tester (non-evolved) population at generation 0 (G0), G30, G60, G100 (see Methods). The ratio of experimental to tester individuals after one full generation of competition provided the data for the estimation of a fitness coefficient, which we term population-wide fitness; see data in [30]. Results from these competition assays were analysed with linear mixed effect models (LMM), with replicate populations taken as a random independent variable and generation as a fixed and continuous independent variable (see Methods).

**Figure 1:**
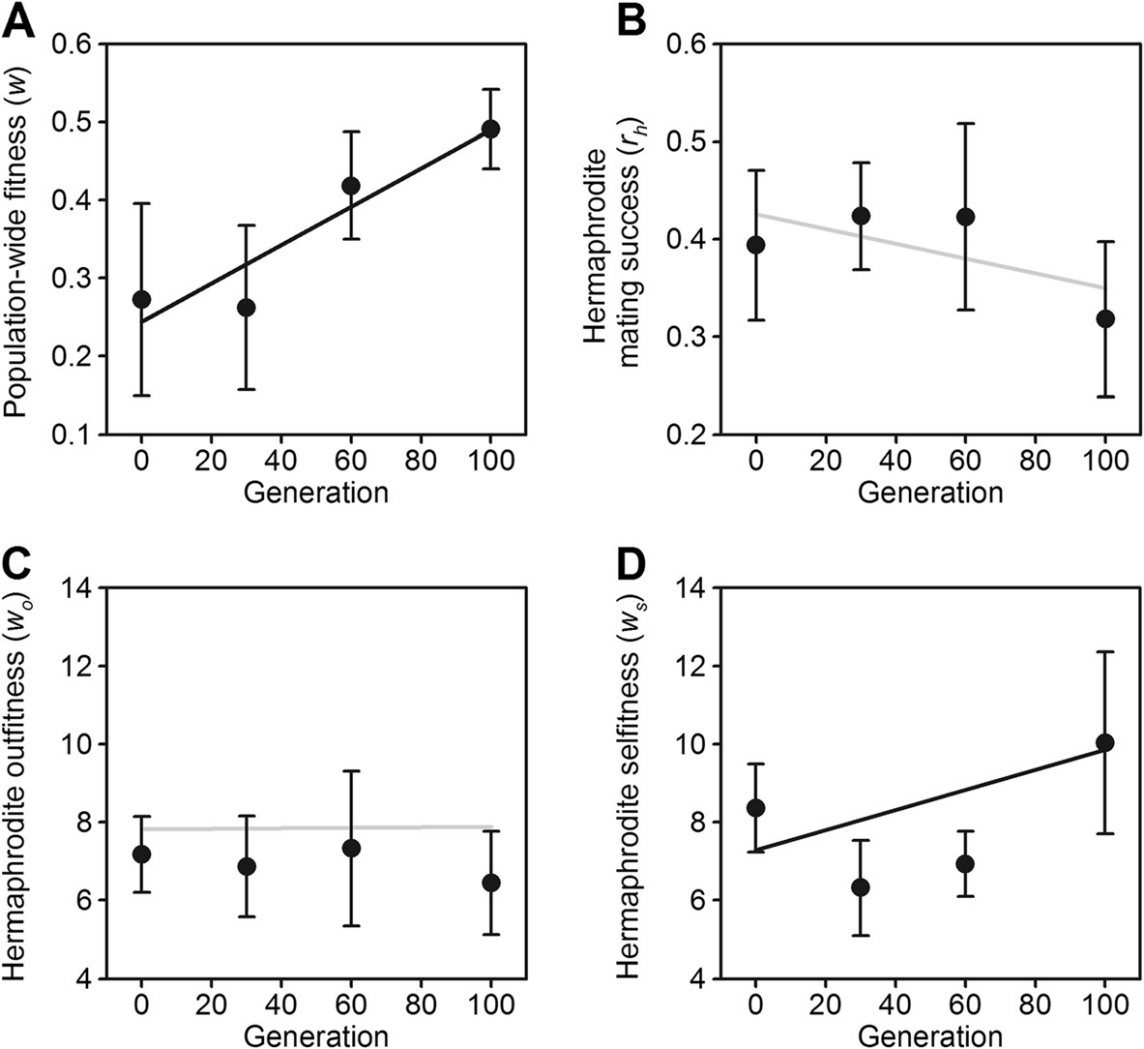
**Adaptation and experimental evolution of hermaphrodite fitness components.** Experimental evolution of population-wide fitness (panel **A**), hermaphrodite mating success **(B)**, hermaphrodite outcross-fitness **(C)** and hermaphrodite self-fitness **(D)**. See Additional file 1: Figure S1, for experimental evolution of outcrossing rates and male fitness, previously reported in ref. [41]. Circles indicate the observed mean values and bars one standard error among the six replicate populations. See Additional file 1 - Supplementary Figure S2 for the observed trajectories of each replicate population. Black straight lines indicate the trend of significant evolutionary responses, and grey straight lines non-significant trends (see text for statistical details).

Analysis shows that population-wide fitness increased during the 100 generations of experimental evolution (Figure 1, panel A; generation effects different from zero: |z|-value = 3, p-value = 0.003, number of observations n = 108). Replicate populations showed heterogeneity in their starting values (accounting for 22% of random genetic variation at G0), and in their dynamics during experimental evolution (Additional file 1: Figure S2).

### Evolution of hermaphrodite mating success and fitness when outcrossing and when selfing

To determine fitness changes in hermaphrodites, we contemporaneously measured the competitive performance of hermaphrodites when selfing or when outcrossing with tester (non-evolved) males at generation 0 (G0), G30, G60, G100 (see Methods). The proportion of outcrossed hermaphrodites within each assay plate was defined as hermaphrodite mating success, while the fertility (number of viable offspring until adulthood) was defined as hermaphrodite fitness under outcrossing or under selfing, depending on the individual breeding status (see data in [30] and Methods). As for population-wide fitness, hermaphrodite mating success was analysed with LMM. For hermaphrodite outcross-fitness and self-fitness data did not follow LMM assumptions and consequently we modelled the effects of generation and replicate population with generalized linear mixed models (GLMM) incorporating Gamma error distributions (see Methods).

LMM analysis indicates that hermaphrodite mating success did not show any evolutionary trend (Figure 1B; generation effects different from zero: |z|-value = 1.1, p-value = 0.46, n = 96). On average, about 40% of the hermaphrodites in each assay plate were outcrossed (intercept = 0.43 ± 0.07SE; |z|-value = 6.3, p-value < 0.001). Replicate population trajectories are shown in Additional file 1: Figure S2.

Hermaphrodite fitness under outcrossing also did not show any trend (Figure 1C; Additional file 1: Figure S2; GLMM generation effects |z|-value = 0.25, p-value = 0.6, n = 215). In contrast, however, hermaphrodite self-fitness increased during experimental evolution (Figure 1D; generation slope |z|-value = 2, p-value = 0.04, n = 328). Significant responses were mostly due to the one of the G100 replicate populations (Additional file 1: Figure S2). Re-analysis without these G100 observations no longer shows a significant evolutionary trend in hermaphrodite self-fitness.

At generation zero there were no differences between hermaphrodite self-fitness (3.6 ± 0.92SE) and hermaphrodite outcross-fitness (2.75 ± 0.62SE). This result suggests that the hybridization with the GFP tester population did not determine the lack of responses because of the expression of outbreeding depression; see also [31].

### Evolution of outcrossing rates and male fitness

Data on outcrossing rates and male fitness (estimated in competition assays in spite of hermaphrodite evolution; see Methods) were reported in [29]. Here, we transformed the original data and re-analysed them with similar LMM as those used for population-wide fitness and hermaphrodite fitness components; see data in [30]. Re-analysis confirmed that outcrossing rates and the male fitness component increased during experimental evolution (Additional file 1: Figure S1).

### Fitness tradeoffs between selfing and outcrossing

With the estimated LMM intercepts for each replicate population we can estimate the genetic correlations among all the traits measured (n = 6; see Methods). We find that population-wide fitness had a negative genetic correlation with hermaphrodite mating success and a positive genetic correlation with hermaphrodite self-fitness (Table 1); thus confirming that they are fitness components. Although being positive in sign, we were unable to detect a significant correlation between population fitness and male fitness, a result that is likely due to the low sample sizes.

**Table 1:** Pairwise correlations among fitness components

	**Hermaphrodite mating success**	**Hermaphrodite outcross-fitness**	**Hermaphrodite self-fitness**	**Male fitness**
Population fitness	-*0.94*	-0.14	*0.94*	0.6
Hermaphrodite mating success	-	0.26	-*0.83*	-0.42
Hermaphrodite outcross-fitness		-	0.14	0.31
Hermaphrodite self-fitness			-	*0.71*

Italic, pairwise Spearman coefficients different from zero under a one-sided test (n = 6; p-values <0.05).

Analysis among fitness components indicates that hermaphrodite mating success had a negative correlation with hermaphrodite self-fitness, but outcross-fitness and self-fitness were not correlated with each other (Table 1). Male fitness and hermaphrodite self-fitness showed a significant positive genetic correlation.

## Discussion

We previously found that populations starting experimental evolution with little genetic diversity evolved increased outcrossing rates, together with increased male fitness, during a period of 100 generations [29]. Here we were interested in finding if outcrossing is adaptive and testing which fitness components could explain the increase in outcrossing rates. Approaching an answer to these two questions would explain why outcrossing can be adaptive in novel environments. Using competitive assays expected to encompass the full life cycle of selection during experimental evolution, we found that population-wide fitness increased during 100 generations, thus indicating that increased outcrossing rates facilitate adaptation. During the same period, hermaphrodite fitness components expressed under selfing may have increased as well but there was little if any evolution of hermaphrodite fitness components expressed under outcrossing. These results suggest that males were the primary drivers in the adaptive evolution of outcrossing.

Correlated evolution between population fitness and male fitness might indicate that sexual selection explains the evolution of outcrossing rates. In particular, sexual selection could have occurred through an increase in either the mating or cross-fertilization success of males [32,33], for example by the evolution of larger sperm [34], or the ability to immobilize hermaphrodites [35]. Consistent with the idea of sexual selection would be the existence of a negative genetic correlation between male fitness and hermaphrodite mating success, as the two sexes have different reproductive interests, cf., [36,37]. We measured such negative correlation between male fitness and hermaphrodite mating success, but found it not to be significant. In addition, and inconsistent with a significant role for sexual selection, there was a positive genetic correlation between hermaphrodite self-fitness and male fitness. These positive correlations suggest that male and hermaphrodite traits were mostly determined by the same loci. The expression of these loci would give little opportunity for the operation of sexual selection, but see [36,38].

Such arguments about the role of sexual selection in the evolution of outcrossing rates are limited to populations that segregate standing genetic diversity or that are subject to the recurrent mutational input of deleterious alleles [31,38-40]. Although conditional on the distributions of fitness effects, it is unlikely that many deleterious alleles accumulated in our experimental evolution. This is because under the life-cycles employed, the population sizes were high enough for reproductive mode to be irrelevant for the efficiency of selection, as we have previously shown in populations starting experimental evolution with standing genetic diversity [41,42].

Interpreting the sign of genetic correlations between fitness components as we did presupposes that mutation rates and their distribution of effects on the several fitness components are equivalent. Unfortunately, there is little empirical data to confirm the validity of this assumption. In *C. elegans*, deleterious recessive mutations tend to be pleiotropic, affecting in a similar direction hermaphrodite life history and morphology [26,43]. Virtually nothing is known about rates of mutation to male traits, though much of the *C. elegans* genome is exclusively expressed in males [44]. Indirect evidence comes from comparing mutation rates among male-hermaphrodite and male–female *Caenorhabditis* species [45]. In this particular study, Baer and colleagues found that lifetime reproduction of males and hermaphrodites/females had a 4-fold higher deleterious mutation rate in male–female species than in male-hermaphrodite species; see also [46,47]. If these comparative results hold at the intra-population level, it is tempting to speculate that in our evolution experiment there was higher mutational variance for male traits than hermaphroditic traits.

Based on the sign of the correlations between hermaphrodite fitness components and population-wide fitness, hermaphrodite self-fitness should have augmented during experimental evolution and hermaphrodite mating success and outcross-fitness should have declined. Yet, there were little evolutionary dynamics in hermaphrodite mating success and fitness. This asymmetry is first justified with the potentially lower number of developmental and behavioural mutational targets in outcrossing traits than selfing traits, as *C. elegans* hermaphrodites are only slightly modified females with a few sperm cells. Second, and perhaps more significantly, since males only reached appreciable frequencies in mid-experimental evolution there was little chance for outcrossing traits to be expressed in hermaphrodites and thus they could not have greatly contributed to population-wide fitness. It is therefore not surprising that population-wide fitness and hermaphrodite self-fitness were positively correlated.

The competitive population-wide assay must have revealed the evolution of hermaphrodite self-fitness. Since the scoring procedure employed in the assay was only based in the presence/absence of GFP expression, we were unable to score for GFP/wild-type heterozygotes and had to assume random mating between the competitors in order to estimate an haploid fitness coefficient, which we took as a surrogate of population-wide fitness. As a result, increased performance under outcrossing will be underestimated since the direction of outcrossing, between GFP males with experimental hermaphrodites and vice-versa, is unknown. The only situation where increased outcrossing performance will not be underestimated is if there was assortative mating within the experimental populations. In this case, homozygous non-GFP genotypes would be more common than expected with genetic drift; see [48] for comparable population-wide competitive assays and results in *C. elegans* populations of different reproduction systems when subject to experimental evolution in novel environments.

Lack of evolution in hermaphrodite fitness when outcrossing, but maybe also when selfing, could indicate that traits expressed under selfing and outcrossing cancelled each other out in their population-wide fitness effects. Congruent with this idea is the fact that hermaphrodite mating success was negatively correlated with both population-wide fitness and hermaphrodite self-fitness. Negative correlations among fitness components in hermaphrodites could in turn explain the reduced influence of hermaphrodites in the evolution of outcrossing rates: specifically, it would explain why the evolution of self-fitness did not prevent the evolution of increased outcrossing rates.

Fitness tradeoffs between selfing and outcrossing might have resulted from the continued antagonistic coevolution between males and hermaphrodites [49,50]. For example, it would have been possible for increased mating ability of males to have countered increased resistance to mating by hermaphrodites. However, there were no negative genetic correlations between male fitness and hermaphrodite mating success, or between male fitness and hermaphrodite outcross-fitness, as would be expected with the antagonistic coevolution scenario. Fitness tradeoffs between selfing and outcrossing might have instead resulted from developmental/ecological resource allocation constraints between male and female functions in hermaphrodites, despite males [51,52]. Congruent with this hypothesis, we found a negative genetic correlation between hermaphrodite self-fitness with hermaphrodite mating success.

Given more time, would outcrossing rates increase to levels close to those found in male–female species? Such outcome is possible, but as soon as males become more frequent there is increased opportunity for the outcrossing traits of hermaphrodites to be expressed and to contribute to population-wide fitness. Hermaphroditic tradeoffs between selfing and outcrossing fitness components could then dominate the adaptive evolution of outcrossing rates, instead of male fitness components. In these circumstances, it could be possible for selfing and outcrossing to be maintained at intermediate frequencies [20].

## Conclusions

In natural *C. elegans* selfing is the predominant mode of reproduction [53], although in the laboratory males are maintained under several environmental and genetic contexts [29,32,54-56]. To our knowledge, only one experimental study has shown that outcrossing in *C. elegans* is adaptive [48]. However, in this study it remained to be shown how within-population variation in male and hermaphrodite traits affected the evolutionary dynamics of outcrossing rates. In contrast, a more recent study has been able to show that male fitness components trading off across generations can maintain outcrossing rates at intermediate levels, despite hermaphrodite fitness components, when *C. elegans* experimentally coevolves with a pathogen [56]. However, whether or not there were correlated changes in population-wide fitness remained unresolved. Our findings therefore confirm that in male-hermaphrodite *C. elegans* increased outcrossing rates evolve because heritable variation in male traits primarily determines adaptation in novel environments.

## Methods

### Experimental evolution

The experimental populations used here were previously described [29]. Six replicate populations (EEViA1-6) were obtained after 11 generations of inbreeding by selfing of hermaphrodites from a population (EEVA0) that ultimately resulted from a pairwise inter-cross among 16 wild isolates. EEViA1-6 were cultured alongside for 100 generations at constant 20°C and 80% RH, under discrete 4 day non-overlapping life-cycles and census sizes of 11030 ± 1438SD (n = 24). (In populations with standing genetic diversity population sizes are an order of magnitude higher than the effective population sizes of Ne = 10^3^; [42]).

The imposed life-cycle during experimental evolution was the following. At day 1, 10^3^ L1-staged worms were seeded in each of ten Petri NGM-lite plates (US Biological) covered with an *E. coli* HT114 lawn that serves as *ad libitum* food. After growth for 3 days, adults from all plates are mixed and killed using a hypochlorite solution and embryos harvested in an hypotonic solution without food. After 24 h, hatched embryos became starvation-arrested L1s, which upon appropriate density estimation were seeded into fresh Petri plates with food to constitute the following generation. This life-cycle was repeated 100 times. Under this scheme, interactions between males and hermaphrodites/females occurred between day 3, when maturity in an average individual is reached, and day 4 of the life-cycle, the day of passaging the population to the following generation. Samples from generation 0 (G0), G30, G60 and G100 were stored at -80°C for posterior contemporaneous characterization [57].

There was no manipulation of male numbers during experimental evolution; cf. [29]. Males initially arose from the spontaneously non-disjunction of the X-chromosome during hermaphrodite gametogenesis at rates of 2×10^-3^ per generation, and sufficient variance in male fitness components must have been subsequently created by mutation for selection to improve their ability to outcross. Otherwise, we would not have been able to measure the concurrent increase in outcrossing rates with male fitness (see re-analysis of this data in Additional file 1: Figure S1). Progeny sex ratios of outcrossed hermaphrodites are on average of 1 male to 1 hermaphrodite [58]. Because there is no sex segregation distortion and hermaphrodites cannot mate with each other, outcrossing rates in a population can be calculated as twice male frequencies, assuming no mixed selfing and outcrossed broods [33].

### GFP tester population

For all the assays reported here, we employed a tester population with a fully penetrant and autosomal dominant *ccls4251(myo3::GFP)* transgenic array. The green fluorescent protein (GFP) expressed by the transgenic array is visible in all muscle cells of larval and adult individuals [59]. The GFP-array was introgressed from strain PD4251 into EEVA0, as previously described [29].

### Population competition assays

Five competitions were done between each experimental population (EEViA1-6) and the GFP-tester population. Generation 0 (G0), G30, G60 and G100 samples were thawed from -80°C stocks and expanded for two generations under similar conditions to avoid confounding environmental effects. On the third generation, 40 experimental and 60 GFP-tester late L4 staged larvae (reproductively immature) were placed together in 6 cm NGM-lite plates with 10 uL of *E. coli*. The proportion of wild-type and GFP males was fixed in this F0 generation (*m*), following the expected average proportion of males among EEViA1-6 observed during experimental evolution; reported in [29] (Additional file 1: Figure S1): for G0, G30, G60 and G100, male proportions were of 0, 0.05, 0.1, and 0.15, respectively. 24 h later, the adults were washed with 200 uL of M9, placed into 6 cm NGM-lite plates and exposed to 200 uL of 1 M KOH: 5%NaOCl for 5 min. Only embryos survive this protocol. 24 h later, L1 larvae were transferred to 9 cm NGM-lite plates with E. coli, with growth pursuing for the next 3 days at 20oC, 80%RH. GFP expression was scored in F1 adults, following standardized transects under a dissection scope at 30x magnification. 306 ± 99SD worms were scored per competition (n = 108).

### Population-wide fitness

To account for the *C. elegans* reproduction system on genotype segregation [45], the observed wild-type frequencies of the F1 generation were corrected to: $p_{F1}=\left(1-2m\right)p+2m\sqrt{p}$; with *m* the male proportion at assay set up (see above), and with *p* the observed frequency of F1 wild-type genotypes. This assumes that hermaphrodites either self- or cross-fertilize, as male sperm outcompetes self-sperm when there is mating [60], and random mating. Population fitness was defined as: $w=\ln\left(\frac{p_{F1}}{1-p_{F1}}/\frac{p_{F0}}{1-p_{F0}}\right)$; with *p*_*F0*_ being the fixed 0.4 wild-type frequencies at F0; cf., [41,61]. *w* measures male and hermaphrodite mating success and their offspring viability. Since there were no males in the competition assays with the generation zero samples, male and hermaphroditic outcrossing traits are not components of fitness in the ancestral populations. However, as males became more frequent during experimental evolution (Additional file 1: Figure S1), variation in male and hermaphroditic outcrossing traits is expected to contribute more and more to fitness.

### Hermaphrodite competition assays

EEViA1-6 population samples were thawed and expanded for three generations in parallel. Per sample, on the fourth generation after thawing, 7 experimental hermaphrodites, 7 GFP-hermaphrodites and 3 GFP-males late L4 staged larvae were placed in competition in 6 cm NGM-lite plates with *E. coli*. 24 h later, 6 experimental hermaphrodites were transferred to NGM-lite plates with *E. coli* and killed with 30uL of 1 M KOH: 5%NaOCl. Three days later, adult worms were scored for GFP expression and number (n = 576). A hermaphrodite was considered outcrossed if it had more than two GFP-male adult offspring.

### Hermaphrodite mating success and fitness

Hermaphrodite mating success (*r*_*h*_) was defined as the relative proportion of hermaphrodites that outcross GFP males at each assay plate (n = 96). Hermaphrodite outcross-fitness (*w*_*o*_) and hermaphrodite self-fitness (*w*_*s*_) hermaphrodite fitness were respectively defined as *r*_*h*_ or (1-*r*_*h*_) times the number of F1 offspring scored per experimental hermaphrodite (n = 215 for outcrossers; n = 328 for selfers). *w*_*o*_ and *w*_*s*_ not only measure hermaphrodite mating and cross-fertilization success but also respective offspring viability until adulthood, despite males.

### Outcrossing rates and male fitness

Assays measuring outcrossing rates and male fitness were reported in [41]; the data is here re-analysed and presented in Additional file 1: Figure S1. We show the male frequencies observed when G0, G30, G60 and G100 samples were measured under the standard experimental evolution environment. Outcrossing rates are be calculated as twice male frequencies (*r* = 2 *m*, [33]). Male fitness assays involved placing an equal proportion of late L4 staged wild type experimental and GFP tester males in competition for mating and fertilization of *fog-2(q71/q71)* females (strain JK574; [62]), during the 24 h preceding the usual passage of experimental evolution. This assay was thus also a head-to-head competition assay, as the assay of population-wide fitness and hermaphrodite fitness. F1 adults were scored for GFP expression (n = 84). Male fitness (*w*_*m*_) is here defined in a similar manner as population fitness (see above), with *p*_*F0*_ = 0.5 and *p*_*F1*_ = *p* since all F1 progeny was outcrossed. Untransformed data was termed male competitive performance in ref. [29]. *w*_*m*_ measures male mating success and male offspring viability, irrespective of hermaphrodites.

### Statistical analysis

We employed linear regression mixed effects models (LMM) to estimate the evolutionary responses of the dependent variables *w*, *r*_*h*_, *w*_*o*_ and *w*_*s*_. For this, generation was taken as a fixed and continuous independent variable and across experimental evolution replicate populations were taken as the random independent variable [63]. A random intercept for each replicate populations and a common fixed slope across experimental evolution were estimated. We re-estimated the evolutionary rates of *r* and *w*_*m*_ with similar LMM modelling (see also Additional file 1: Figure S1); but see Figure six and Table one in [29].

Alternative modelling of random replicate effects at each generation would provide inconsistent estimates of the fixed generation effects [64], as with only six replicates there is no power to model heterogeneity among populations (no matter how many measurements per replicate were collected). There was no nesting of competition assay plate within replicate population or replicate population within generation, and we thus assume that the variance among competition plates is the same for all populations and is maintained constant during experimental evolution.

For all models we tested the residuals for normality with Shapiro-Wilk tests and for homocedasticity between the four generations with Bartlett’s tests. *w*_*o*_ and *w*_*s*_ data violated LMM assumptions and because of this we resorted to generalized linear mixed effects models (GLMM) with Gamma error distributions. Only in this case we were able to have normally distributed residuals with equal replicate variance between generations.

For LMM and GLMM, REML methods were used within the *lme4* package of R version 3.0.2 [65,66]. To obtain fitted values in the original scale, particularly for *w*_*o*_ and *w*_*s*_, we used the function *predict* within the *stats* package in R. For significance of replicate population effects (intercept of the models) and generation (slope) from zero, we employed z-tests as implemented in the *multcomp* R package *cftest* function [67].

### Correlations among the traits measured with competition assays

The fitted intercepts of the LMM or GLMM are an estimate of the random genetic differences between the six replicate populations, despite the experimental evolution responses due to mutation. As long as one of the independent variables in LMM or GLMM is significantly different from zero (whether the average replicate population effect at G0 or generation effects) fitted values can be taken for posterior analysis. We use the fitted intercepts to estimate genetic correlations among all the traits measured here.

To determine which sex-specific traits are fitness components we calculated the Spearman’s rho rank correlation coefficients between the fitted intercepts of *w* with the fitted intercepts of *r*_*h*_, *w*_*o*_, *w*_*s*_, *w*_*m*_. To determine whether fitness tradeoffs between selfing and outcrossing underlie adaptation we calculated the pairwise Spearman’s coefficients between *r*_*h*_, *w*_*o*_, *w*_*s*_, *w*_*m*_. The total sample size was of n = 6, corresponding to the six starting G0 replicate population LMM/GLMM estimates. We used the *cor.test* function within the *stats* package in R to calculate rho and significance. We cannot calculate the correlation between population-wide fitness (*w*) and outcrossing rate (*r*) because at G0 *r* ≈ 0. Our test for outcrossing being adaptive is based on the LMM significance of generation effects in *w*.

### Availability of supporting data

The data sets supporting the results of this article are available in the Dryad.org repository, doi:10.5061/dryad.h6231.

## Competing interests

The authors declare that they have no competing interests.

## Authors’ contributions

SC, CG and HT conducted experimental evolution and did the population competition assays. IMC and HT did the hermaphrodite competition assays, analyzed the data and wrote the manuscript. All authors read and approved the final manuscript.

## Supplementary Material

Additional file 1 **Supplementary Figures.** This PDF file contains Supplementary Figure S1 with the experimental evolution of outcrossing rates and male fitness. It also contains Supplementary Figure S2 with the replicate experimental evolution trajectories of population fitness, hermaphrodite mating success, hermaphrodite outcross-fitness and hermaphrodite self-fitness. Click here for file
