# Supplementary material for: The role of hermaphrodites in the experimental evolution of increased outcrossing rates in Caenorhabditis elegans
Source: BMC Evol Biol. 2014 Jun 2;14:116. doi: 10.1186/1471-2148-14-116 (PMC4055231; doi:10.1186/1471-2148-14-116)
Supplement: Additional file 1 — Supplementary Figures. This PDF file contains Supplementary Figure S1 with the experimental evolution of outcrossing rates and male fitness. It also contains Supplementary Figure S2 with the replicate experimental evolution trajectories of population fitness, hermaphrodite mating success, hermaphrodite outcross-fitness and hermaphrodite self-fitness. [file 1471-2148-14-116-S1.pdf]

Additional File 1 – Supplementary Figures 1 and 2 for:

The role of hermaphrodites in the Caenorhabditis elegans experimental evolution of increased outcrossing rates

Authors: Sara Carvalho, Ivo M. Chelo, Christine Goy and Henrique Teotónio

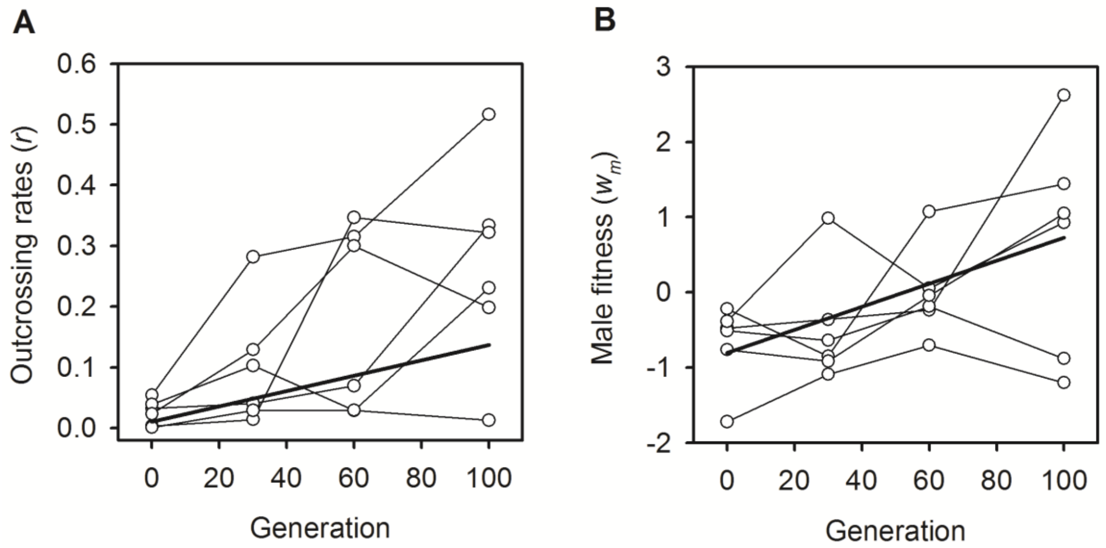

Supplementary Figure 1. Experimental evolution of outcrossing rate (panel A) and male fitness (B). The data was previously reported in [41], but is here transformed and re-analyzed (see main text). Circles and connecting lines indicate the average replicate population trajectories. Black straight line indicates the trend of significant evolutionary responses (see main text for statistical modeling methods). For outcrossing rates LMM indicated a significant generation effect (|z|-value=6.7, p-value<0.001, n=44) and no differences at G0 from zero (intercept |z|-value=0.59, p-value=0.56). For male fitness LMM indicated a significant generation effect (|z|-value=5.4, p-value<0.001, n=84). For both traits, normality of residuals and homocedasticity was followed.

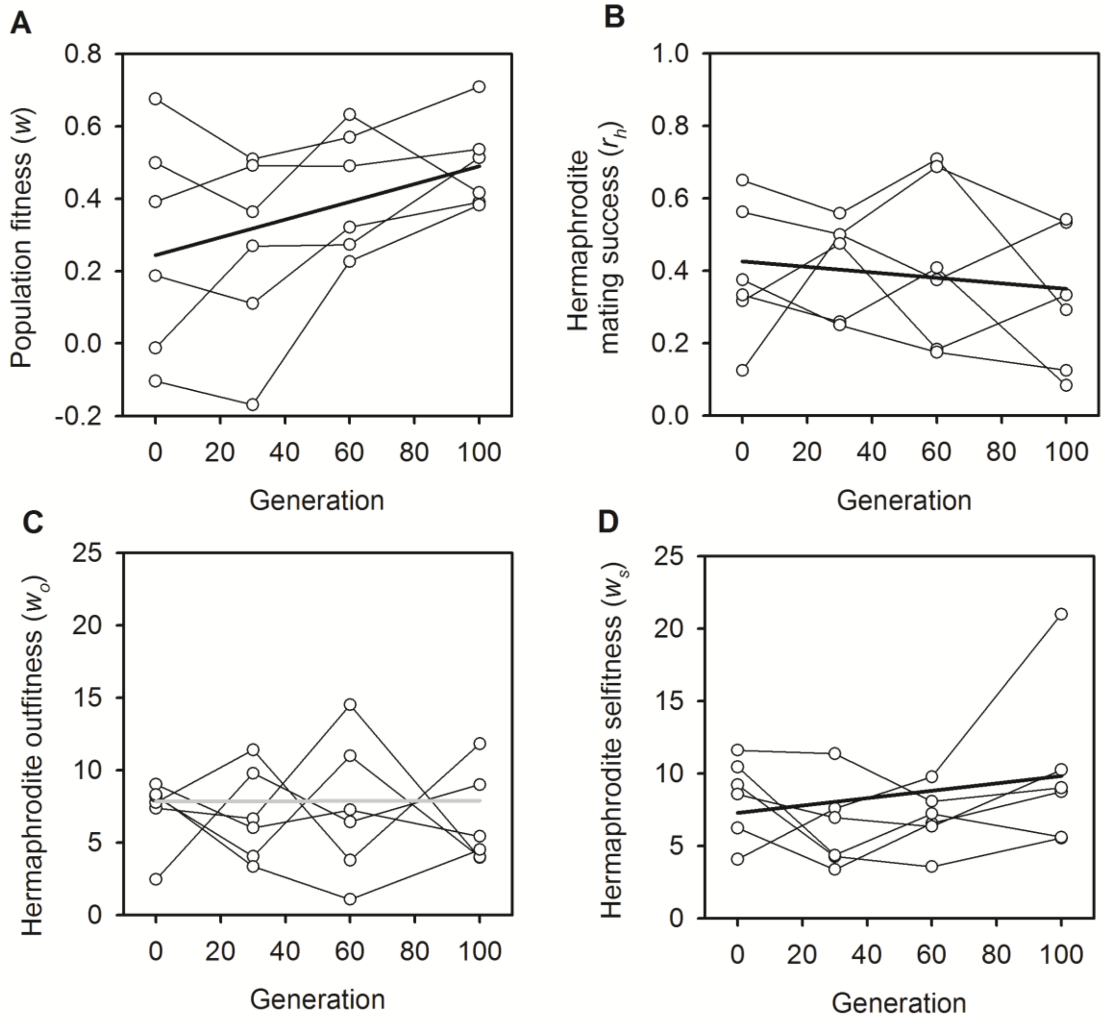

Supplementary Figure 2. Experimental evolution of population-wide fitness (panel A), hermaphrodite mating success (B), hermaphrodite outcross-fitness (C), and hermaphrodite self-fitness (D). Circles and connecting lines indicate the average replicate population trajectories. Black straight line indicates the trend of significant evolutionary responses, and grey straight line non-significant trends.
